# Advancements in Composite Materials and Their Expanding Role in Biomedical Applications

**DOI:** 10.3390/biomimetics8070518

**Published:** 2023-11-01

**Authors:** Sivakamavalli Jeyachandran, Hethesh Chellapandian, Nemat Ali

**Affiliations:** 1 Lab in Biotechnology & Biosignal Transduction, Department of Orthodontics, Saveetha Dental College & Hospitals, Saveetha Institute of Medical and Technical Sciences (SIMATS), Saveetha University, Chennai 600077, India; 2 Department of Pharmacology and Toxicology, College of Pharmacy, King Saud University, P.O. Box 2457, Riyadh 11451, Saudi Arabia

**Keywords:** biopolymer, Ni-doped ZnO, nanocomposite, band gap, anticancer activity

## Abstract

The synthesis of a Ni-doped ZnO nanocomposite incorporating chitosan (CS/Ni-doped ZnO) was achieved via a precipitation method, followed by annealing at 250 °C. This study comprehensively examined the nanocomposite’s structural, functional, morphological, and porosity properties using various analytical techniques, including X-ray diffraction (XRD), Fourier transform infrared spectroscopy (FTIR), high-resolution scanning electron microscopy (HR-SEM), transmission electron microscopy (TEM), and Brunauer–Emmett–Teller (BET) analysis. The presence of chitosan (CS) and nickel (Ni) within the nanocomposite, along with their influence on reducing the band gap of ZnO particles and enhancing the generation of electron-hole pairs, was confirmed using UV-visible near-infrared spectroscopy (UV-vis-NIR). The electrochemical properties of the CS/Ni-doped ZnO nanocomposite were investigated via electrochemical impedance spectroscopy (EIS) and cyclic voltammetry (CV) by utilizing a phosphate buffer solution with a pH of 6, which closely resembled the typical pH of bacterial cell walls. Finally, the prepared CS/Ni-doped ZnO nanocomposite was evaluated for its antibacterial and anticancer activities. The results demonstrated the highest inhibition of bacterial growth in *P. vulgaris*, whereas the lowest inhibition was found in *S. aureus* across various concentrations, thus highlighting its potential in antimicrobial applications. The cytotoxicity of CS/Ni-doped ZnO nanocomposites demonstrated remarkable effects with a half-maximum inhibitory concentration of approximately 80 ± 0.23 µg mL^−1^ against MCF-7 breast cancer cell lines, following a dose-dependent manner.

## 1. Introduction

Recently, the construction of hybrid structure-based nanoparticles has become a very hot topic in the biomedical, material science and engineering fields due to their unique properties [1]. They consist of organic and inorganic materials with at least one component in nanodimensions [2]. In the development of hybrid nanostructures, the utilization of natural organic biopolymers of chitosan is of significant interest as the primary source. In this regard, inorganic metal oxides such as ZnO, TiO_2_ and NiO nanoparticles are used extensively in the fabrication of antibacterial agents to enhance the sterile nature of the materials [3,4,5].

Chitosan is a naturally occurring biopolymer, which is deacetylated from chitin soluble only in weak acids, such as acetic and formic acids [6], and maximum deacetylation (70% to 98%) is obtained from the exoskeleton and cell walls of crustaceans [7,8]. It contains high units of hydroxyl and amino functional groups, which provide several side chains that are stable in the highly hydrophobic natures of both acidic and basic mediums. The presence of OH and NH_2_ functional groups are used to stabilize metal oxide nanoparticles and interact with the bacterial membrane of the negative charge containing phospholipids and proteins, which is essential for antibacterial effects in acidic conditions [9] and exhibits self-imbibed biocompatibility, nontoxicity and eco-friendliness [10,11,12].

Additionally, zinc oxide (ZnO~3.3 eV) is a semiconducting metal oxide with transparency in visible light and exhibits superior antibacterial activity [11,12,13,14,15,16,17]. Moreover, material science researchers suggested that ZnO is highly effective in antibacterial activity with a pH range of 6-8 [18] and has three features: first, ZnO releases the dissociated Zn^2+^ [19,20]; second, ZnO interacts with the surface of the bacterial cell wall [21]; third, reactive oxygen species (ROS) is generated, such as oxygen anions, hydrogen peroxide, hydroperoxides and hydroxyl radicals [22]. However, some limitations, principally the fast recombination rate of the generated electron and positive hole, occur in pure ZnO nanoparticles used as antibacterial agents [23]. To overcome these limitations, ZnO is doped with different metallic ions such as Ce, Ag, Fe and Ni [13,24,25,26,27]. Among the metal ion oxides, nickel oxide (NiO) is a p-type material with an excitation energy of 110 MeV and favorable electronic structure and optical properties. It is capable of acting as a good transporting agent that is nontoxic and eco-friendly [28,29,30]. Several researchers reported the photodegradation of organic dyes with the sol–gel process using Ni-doped ZnO nanospheres [31], Ce–ZnO composites using the microwave irradiation method [32] and the co-precipitation route using Ni-doped ZnO nanorods [33]. The microwave-assisted synthesis of the Ag–ZnO nanocomposite was used to examine antibacterial activity against *Staphylococcus aureus* and *Escherichia coli* [34] and the hydrothermal reaction of zinc-doped nickel hydroxide nanosheets in a nonenzymatic glucose sensor [35]. These results indicate that different metal ions can be doped with ZnO interstices to improve their properties for various applications. Other researchers used chitosan-based hybrid nanocomposites of chitosan–ZnO [36], chitosan/Ni_0.2_Zn_0.2_Fe_2.6_O_4_ [37], chitosan-Au/Pd/Ag NPs [38], chitosan/Ag/MoS [39], chitosan–Ni/NiO [40] and Ni/Al/Chitosan [41] for phocatalysis, as well as biomedical and electrochemical applications. Here, we develop the synthesis of biogenic chitosan incorporated with Ni-doped ZnO nanocomposites, and its biocompatible properties are checked using antimicrobial and anticancer activities. In the first step, the Ni-doped Zn(OH)_2_ nanocomposite was synthesized via a chemical precipitation route, after which chitosan was functionalized with the Ni-doped Zn(OH)_2_ nanocomposite using a hydrothermal process. The addition of chitosan and Ni doping to the ZnO nanostructure was systematically studied, and the crystalline, structural, morphological and electrochemical properties were investigated using various techniques. Further, the microbial inhibitory activity of the nanocomposite was evaluated against bacterial strains including both Gram-positive and -negative bacteria, and in vitro anticancer activity was assessed in MCF-7 breast cancer cell lines.

## 2. Experiments

### 2.1. Materials

Chitosan (molecular weight of 310 kDa, degree of deacetylation: 80–95%) was purchased from South India Seafoods, Rameswaram, Tamil Nadu, India. Analytical grade nickel nitrate hexahydrate [Ni(NO_3_)_2_.6H_2_O, 99%] and zinc nitrate hexahydrate [Zn(NO_3_)_2_.6H_2_O, 99.8%] were purchased from LOBA Chemie (P) Ltd., Mumbai, India. Sodium hydroxide (NaOH, 98%) was purchased from CDH (P) Ltd. Analytical grade disodium phosphate (Na_2_HPO_4_) and monosodium phosphate(NaH_2_PO_4_) were purchased from LOBA Chemie (P) Ltd., Mumbai, India. Acetic acid (CH_3_COOH, 99.7%), acetone (CH_3_CHO, 99.9%) and ethanol (C_2_H_5_OH, 99.8%) were purchased from Fischer Chemic Ltd., India. The chemicals were of an analytical reagent grade and used without any further purification. Millipore water was used in all experiments.

### 2.2. Synthesis of Nickel-Doped Zn(OH)_2_ Composite

**Scheme 1:**
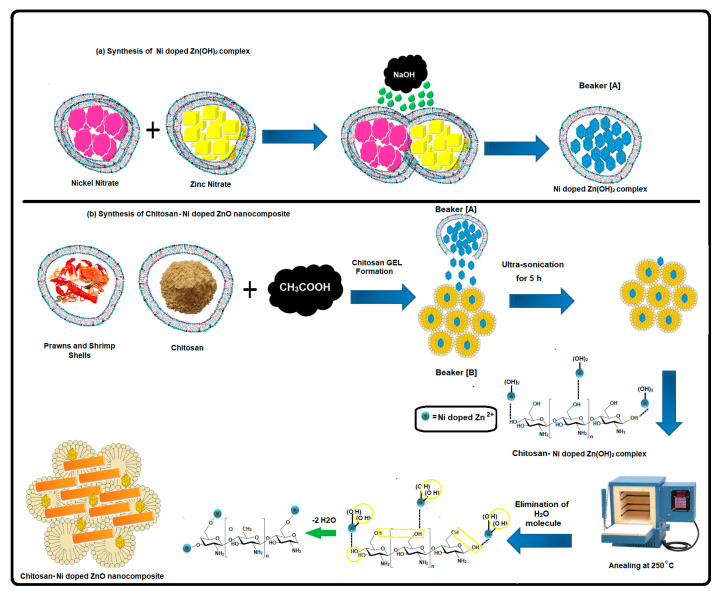
Schematic representation of Ni doped Zn(OH)_2_ complex preparation (**a**) Polymeric chitosan with Ni doped ZnO nanocomposites (**b**).

The Ni-doped Zn(OH)_2_ composite was synthesized using a precipitation method, and the schematic representation is illustrated in Scheme 1a. About 1M of Zn(NO_3_)_2_.H_2_O and 0.25 M of Ni(NO_3_)_2_.6H_2_O were dissolved in 50 mL of deionized water under magnetic stirring for 40 min at room temperature. Then, 3M of NaOH was added dropwise into the above mixture and stirred for 1 h until the complex precipitate was obtained. The obtained product was rinsed multiple times with deionized water and ethanol. It was designated as beaker A. 

### 2.3. Synthesis of CS/Ni-Doped ZnO Nanocomposite

The synthesis of the CS/Ni-doped ZnO nanocomposite was carried out using a two-step chemical precipitation method, which is illustrated in Scheme 1b. In the first step, chitosan (25 mg) was dissolved in a 50 mL volume of 3% acetic acid under magnetic stirring for 1 h at room temperature (28 °C) to form a gel phase solution, the pH was adjusted to 8, and the solution was designated as beaker B. Second, the prepared Ni-doped Zn(OH)_2_ complexes (beaker A) were slowly added into the dispersed chitosan (beaker B). Then, an ultrasound was used on the reaction mixture for 5 h to form a Ni-doped Zn(OH)_2_ dispersed chitosan matrix. During this process, the bulk Ni-doped Zn(OH)_2_ particles were exfoliated and bound with the functional group of the chitosan matrix. Finally, the reaction mixture was heated in an oven at 120 °C for 2 h. The ZnO, binary composites of CS/ZnO and Ni-doped ZnO were synthesized using a similar procedure. Finally, the obtained products were heated in a hot air oven at 250 °C for 3 h with a ramp rate of 10 °C min^−1^. The synthesis of the CS/Ni-doped ZnO nanocomposite is clearly depicted in Scheme 1.

### 2.4. Fabrication of CS/Ni-Doped ZnO Nanocomposite Modified GCE

Before modification, the glassy carbon electrode (GCE) was refined using a 0.05μm alumina slurry and then rinsed thoroughly with distilled water under an ultrasound for 5 min and dried in the air. The CS/Ni-doped ZnO nanocomposite (1 mg) was dissipated in 2 mL of ethanol under an ultrasound for 30 min, after which 5 μL of the suspension was dropped onto the surface of the pre-cleaned GCE using the drop-casting method. Finally, the modified electrode was dried in the air for 2 h at room temperature.

### 2.5. Characterization

The crystalline behavior of the materials was studied using the X-ray diffraction (XRD) patterns obtained from an X-ray diffractometer (model XPERT-PRO) (Rigaku diffractor with CuKα radiation) with ambient conditions over a 2θ region of 20–80° at a rate of 2°/min (40 kV, 20 mA). The morphological studies were obtained via HR-SEM (HR-SEM, FEI-quanta FEG 250) and TEM (FEG-TEM 300 kV) analyses. The structural properties were measured using the FT-IR spectrum and recorded on a PerkinElmer 2000 spectrophotometer, with a range of 4000–400 cm^−1^, using KBr pellets at room temperature. The optical properties of the samples were examined using UV-Vis-NIR spectroscopy (JASCO spectrophotometer). The specific surface area and pore volume of the synthesized products were measured using the nitrogen adsorption/desorption isotherm with Gemini model 2380. The electrical properties of the samples were measured using a CH instrument (Autolab model CHI1102A).

The voltammetric and electrochemical impedance spectroscopy (EIS) studies were carried out using a three-electrode system containing a 50 mM phosphate buffer solution (pH = 6) of 5 mM [Fe (CN)_6_]^3−/4−^ with a potential range from 0.2 to 0.8 V and AC frequency range from 10^5^ kHz to 0.01 Hz at OCP with an amplitude of 5 mV. Glassy carbon (3 mm diameter), platinum and saturated calomel electrodes were used as working, counter and reference electrodes, respectively.

### 2.6. Antimicrobial Evaluations

The antibacterial activities of the prepared CS/Ni-doped ZnO nanocomposite were tested against Gram-positive and -negative bacteria, as shown in Table 1. The zone of inhibition was measured using the well diffusion method, and the effect was compared with that of a commonly used antibiotic like Ampicillin. The abovementioned bacteria were grown individually. The nutrient agar medium was used for bacterial growth and was poured onto Petri plates. Fresh bacterial cultures of both organisms were swabbed onto the agar medium and incubated at 37 °C for 24 h. The antimicrobial activity was evaluated by measuring the zone of inhibition.

### 2.7. Cytotoxicity Studies

The cytotoxicity (in vitro) study was conducted with the CS/Ni-doped ZnO nanocomposite at different concentrations (0–150 µg mL^−1^) against Hep G-2 human liver cancer cell lines based on the methodology adopted by Mosmann et al. (1983) [42]. In the end, the IC50 concentration of the CS/Ni-doped ZnO nanocomposite was calculated using Origin Pro 8 software. Further, the IC50-treated liver cancer cell lines were visualized for morphological changes using an inverted phase contrast microscope.

## 3. Results and Discussion

### 3.1. XRD Analysis

**Figure 1:**
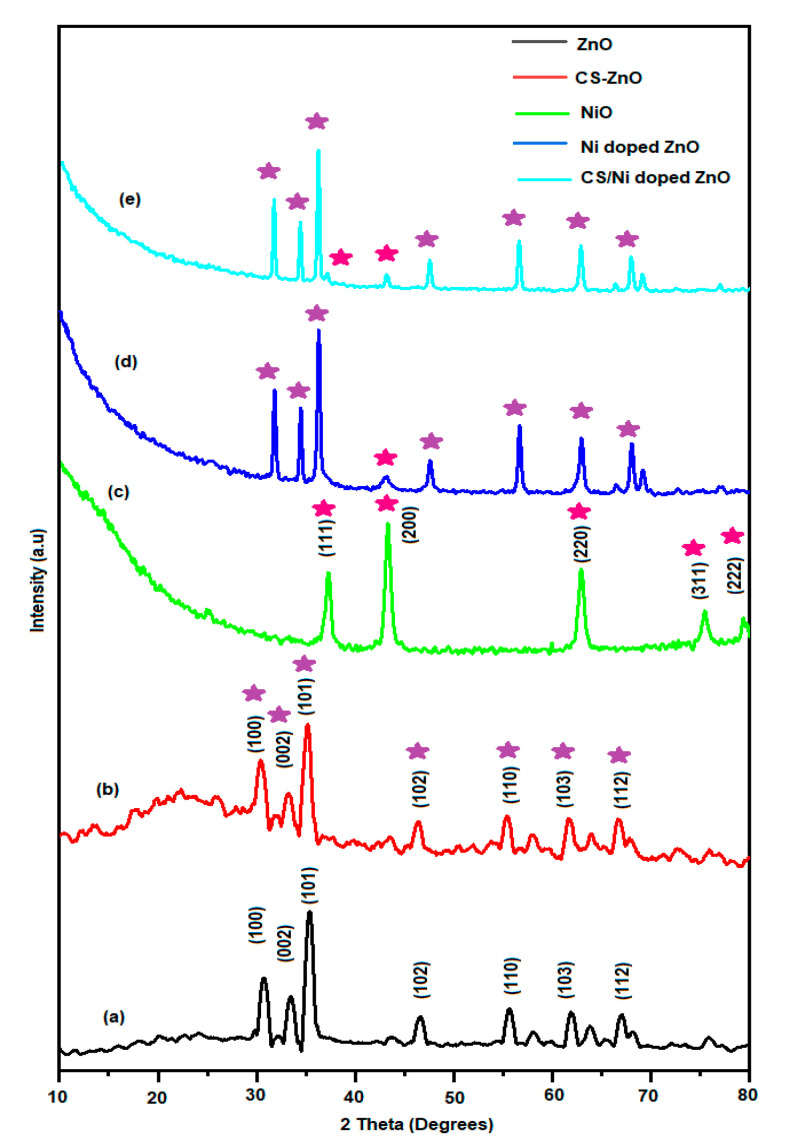
XRD patterns of (**a**) the pristine ZnO, (**b**) CS/ZnO binary composite, (**c**) pristine NiO, (**d**) Ni-doped ZnO and (**e**) ternary hybrid nanostructure of the CS/Ni-doped ZnO.

The crystalline structure of pristine ZnO and NiO, the binary structure of CS/ZnO and Ni-doped ZnO and the ternary structure of the CS/Ni-doped ZnO nanocomposite are shown in Figure 1. The pristine ZnO (Figure 1a) displays the characteristic peaks appearing at 30.83, 33.48, 35.42, 46.30, 55.73, 61.54 and 66.61° and corresponding to the planes of hexagonal wurtzite ZnO (100), (002), (101), (102), (110), (103) and (112), which are consistent with the JCPDS card numbers 01080-0075, respectively. The CS/ZnO binary composite (Figure 1b) clearly displays that the plane of (002) decreased in intensity, which indicates that the ZnO nanoparticles are incorporated with the chitosan matrix [32]. Meanwhile, the pristine NiO (Figure 1c) exhibits characteristic peaks at 37.11, 43.40, 62.98, 75.31 and 79.43° corresponding to the planes of (111), (200), (220), (311), and (222), respectively, which accord with the cubic crystal structure of NiO nanoparticles [43]. The crystal pattern of the Ni-doped ZnO (Figure 1d) exhibits a new peak at 2θ = 43.40°, which corresponds to the plane of nickel oxide (200), and the intensity of the ZnO planes increases, which may be attributed to the smaller ionic radius of Ni^2+^ ions (ionic radius = 0.69 nm) easily substituted at Zn^2+^ sites (ionic radius = 0.74 nm). Generally, the replacement of the host metal ion by the guest metal takes place when the size of the dopant ion is of reduced radius size than that of the guest lattice ion [44], which suggests that nickel ions are incorporated into the ZnO lattice. The ternary hybrid nanostructure of the CS/Ni-doped ZnO is shown in Figure 1e. A narrow peak intensity and a new peak appear at 2θ = 37.11 and 43.40° corresponding to the planes of nickel oxide (111) and (200), which are ascribed to the chitosan and act as a metal ion chelating agent. Meanwhile, the addition of a lower ionic radius of Ni^2+^ creates intrinsic stress on the ZnO lattice sites. The biogenic chitosan and Ni^2+^ ions altered the sizes, shapes and microstructures of the ZnO nanoparticles. 

### 3.2. FTIR Analysis

**Figure 2:**
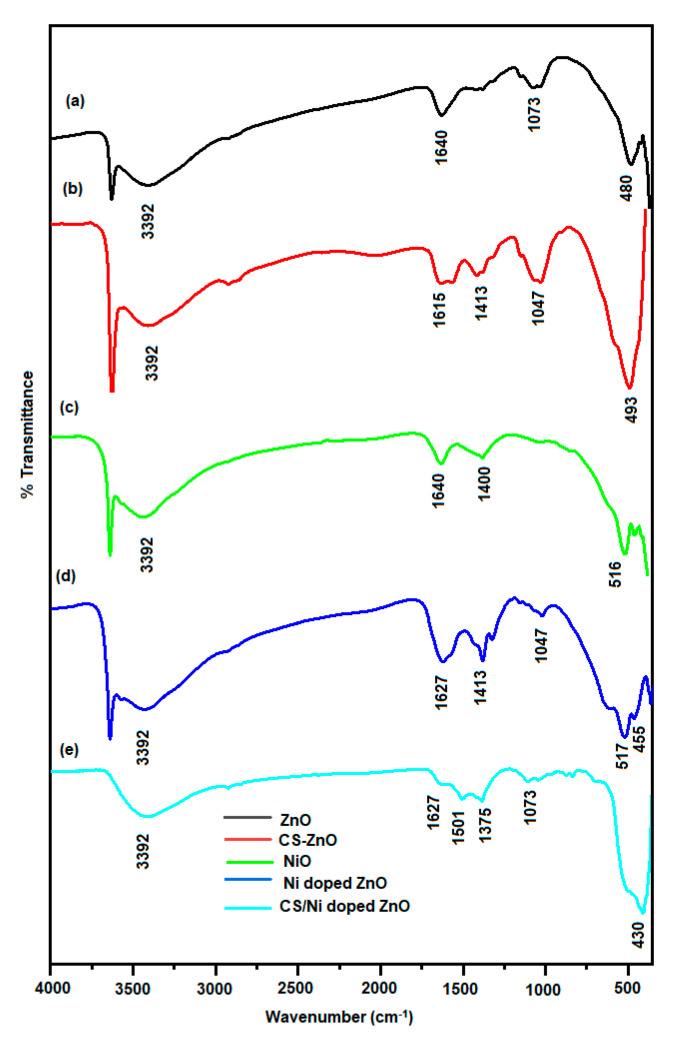
FTIR spectra of (**a**) ZnO, (**b**) CS-ZnO, (**c**) NiO, (**d**) Ni-doped ZnO and (e) CS/Ni-doped ZnO.

The structural properties of the synthesized materials were analyzed using the FT-IR spectra and the results are illustrated in Figure 2. As per this study, pristine ZnO (curve a) displays have characteristic peaks at 3421, 1640, 1073 and 480 cm^−1^ representing the O-H stretching, H-O-H bending, Zn-O-Zn stretching and formation of the Zn-O bond, respectively [30,33,45]. The chitosan-incorporated ZnO (Figure 2b) exhibits peaks at 3392, 1615, 1413, 1047 and 493 cm^−1^ corresponding to the O-H stretching, amine group (-NH_2_) bending, symmetrical deformation of the CH_3_ group, C-O-C stretching of saccharide units and ZnO stretching vibrations, respectively [46,47]. The major characteristic peaks of pristine NiO (curve c) appear at 1640 and 516 cm^−1^ corresponding to the H-O-H bending and Ni-O vibrations, which indicates the presence of NiO [48]. The additional peak at 1400 cm^−1^ may be ascribed to the nitrate (NO_3_-) group, which arises from the source material of nickel nitrate [49]. When the results of the Ni-doped ZnO (curve d) are compared with the pristine ZnO (curve a), two distinct broad peaks are seen at around 517 and 455 cm^−1^ representing Ni-O and Zn-O, respectively, and certain peaks are moved slightly to lower and higher wave numbers indicating that Ni^2+^ has been occupied at Zn^2+^ sites. The FTIR spectra of CS/Ni-doped ZnO nanocomposite (curve e) have characteristic peaks at 3392, 1627, 1501, 1375, 1073 and 430 cm^−1^ corresponding to the O-H stretching, bending vibration of NH_2_, C-N vibration, CH_3_ symmetrical deformation, C-O-C stretching of saccharide units and Ni-doped ZnO, respectively, which indicates that the lower ionic radius of guest Ni^2+^ ions strongly influenced the host ZnO and created a lattice defect in the chitosan matrix by intrinsically binding a large number of metal oxides into the host zinc oxide sites. Also, the synergistic effect of the three different units creates shifts in peak positions. 

### 3.3. UV-Vis-NIR Analysis

**Figure 3:**
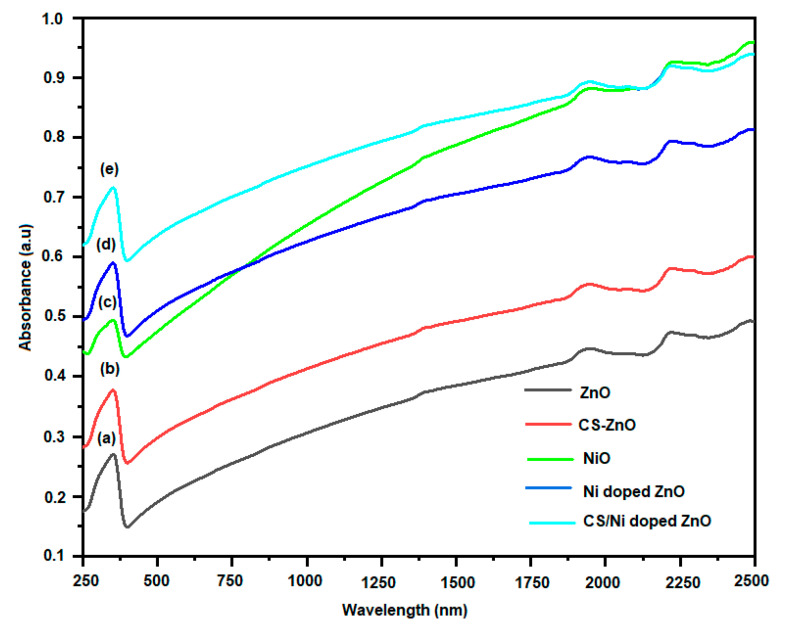
The UV-Vis-NIR spectra peak of (**a**) ZnO, (**b**) CS/ZnO, (**c**) NiO, (**d**) Ni-doped ZnO and (**e**) CS/Ni-doped ZnO.

The UV-Vis-NIR spectra of ZnO, CS/ZnO, NiO, Ni-doped ZnO and CS/Ni-doped ZnO nanocomposite in the wavelength range of 250-2500 nm are shown in Figure 3. All the samples have strong absorption spectra at the UV region between 250 and 375 nm. The UV-Vis-NIR spectra peak of ZnO (Figure 3a), CS/ZnO (Figure 3b), NiO (Figure 3c), Ni-doped ZnO (Figure 3d) and CS/Ni-doped ZnO (Figure 3e) nanocomposites correspond to 343, 348, 349, 353 and 356 nm, respectively. The result of the CS/Ni-doped ZnO nanocomposite spectrum is slightly red and shifted to a higher wavelength compared to the Ni-doped ZnO.

**Figure 4:**
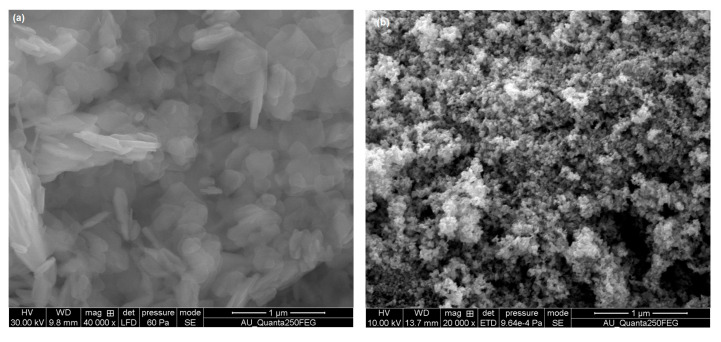

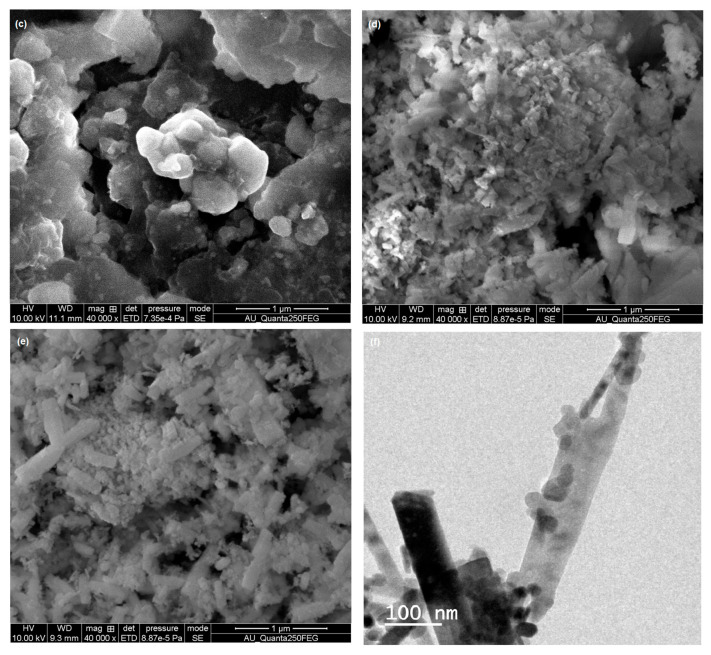
SEM images: (**a**) pristine ZnO; (**b**) aggregated NPs and CS/ZnO; (**c**) particles that also exhibit agglomerated grain structure; (**d**) Ni-doped ZnO; (**e**) ternary structure of CS/Ni-doped ZnO nanocomposite; (**f**) 100 nm magnification of CS/Ni-doped ZnO nanocomposite exhibits a rod-like structure.

This may be due to the synergistic effect of organic and inorganic material increasing the wavelength. In addition, the biosurfactant of the biogenic chitosan polymer was intrinsically coupled with the metal oxide particles and increased the wavelength. The band gaps of these absorptions are shown in Figure 4. The band gaps are calculated as described in previously published papers [50,51]. As shown in Figure 4, the band gap energies for these samples decreased from 3.17, 3.05, 2.92, 2.86 and 2.79 eV for ZnO (Figure 4a), CS/ZnO (Figure 4b), NiO (Figure 4c), Ni-doped ZnO (Figure 4d) and CS/Ni-doped ZnO (Figure 4e) nanocomposites, respectively. It was concluded that the incorporation of biogenic chitosan led to a decrease in the optical band gap of the CS/Ni-doped ZnO nanocomposite.

Furthermore, the comparable ionic radius of Ni^2+^ also reduced the band energy, which is generally attributed to the Burstein–Moss shift and resulted in filled electronic states near the bottom of the conduction bands [52,53].

### 3.4. Morphological Analysis

The surface morphologies of the synthesized materials are displayed in Figure 4. From these pictures, it can be seen that pristine ZnO (Figure 4a) nanoparticles have aggregated particles and CS/ZnO (Figure 4b) exhibits a porous structure, where the metal oxides have larger embedded micropores that could be ascribed to the electrostatic interactions between the chitosan backbone and ZnO nanoparticles [54]. Figure 4c displays the particles, which also exhibit agglomerated grain structures. The Ni-doped ZnO (Figure 4d) displays an agglomerated grain structure due to Ni^2+^ incorporated into ZnO, and the defect of lattice distortion is induced by Ni^2+^ in Zn-O sites [55,56]. Also, it was observed that the cubic shape and nonuniform size and agglomeration of secondary NiO particles were seen due to the small dimensions and high activation energy of metal oxide particles [55,56]. The ternary structure of the CS/Ni-doped ZnO nanocomposite (Figure 4e) depicts a rod-like structure, which was assigned to the ZnO nanoparticles and intrinsically distributed in the chitosan biopolymer, absorbing a large number of comparable ionic radii of nickel metal ions that contain metal hydroxides at ZnO sites. Compared to the binary structure of the Ni-doped ZnO composite, the ternary structure of the CS/Ni-doped ZnO nanocomposite shows a more packed and well-shaped structure, which may be due to the chitosan polymer extending the growth rate of metal oxide and producing a rod-shaped structure. Finally, the ternary structure of the CS/Ni-doped ZnO nanocomposite analyzed using TEM and displayed in Figure 4f exhibits a rod-like structure with a range of 100 nm, which is consistent with the SEM results.

### 3.5. BET Analysis

**Figure 5:**
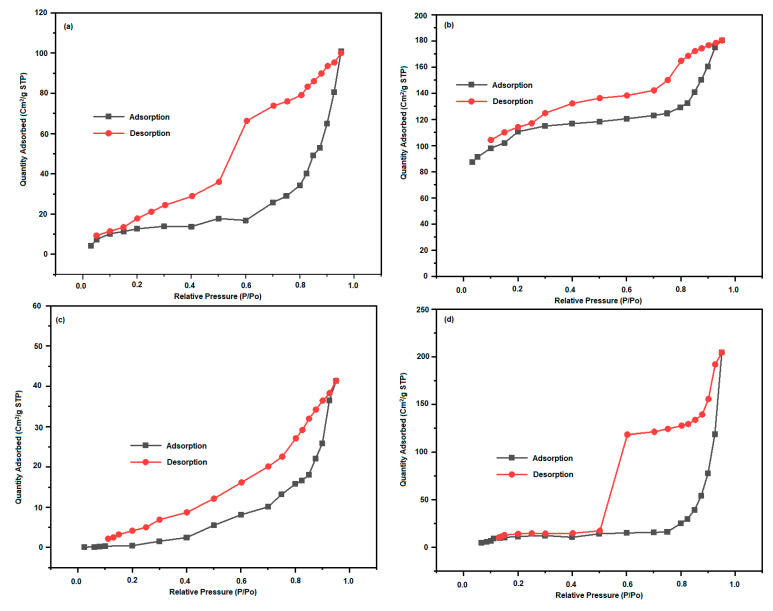

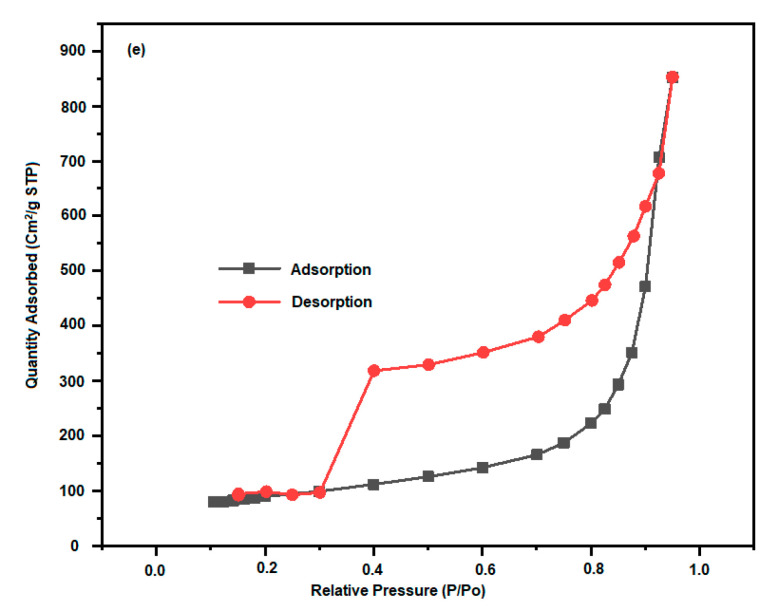
The synthesized materials of (**a**) ZnO; (**b**) CS/ZnO; (**c**) NiO; (**d**) Ni-doped ZnO; (**e**) CS/Ni-doped ZnO nanocomposite were measured using nitrogen adsorption–desorption isotherms.

The synthesized materials of ZnO, CS/ZnO, NiO, Ni-doped ZnO and CS/Ni-doped ZnO nanocomposite were measured using nitrogen adsorption–desorption isotherms and are shown in Figure 5, and the results of all samples have type IV characteristics, suggesting their mesoporous features [57]. Table 1 shows the calculated surface areas, pore volumes and pore sizes of the materials. From these results, the ternary structure of the CS/Ni-doped ZnO nanocomposite has a larger surface area due to the doping level of Ni into the ZnO lattice sites making some changes and defects in the crystalline microstructure, leading to the enhancement of the porous character [58]. as Additionally, the porous nature of the chitosan acts as a stabilizing agent, and it could be embedded within the pores present in the shells of the ZnO particles, thus creating some defects and improving the surface area and porosity character of the nanocomposites.

### 3.6. Electrochemical Impedance Spectroscopy

**Figure 6:**
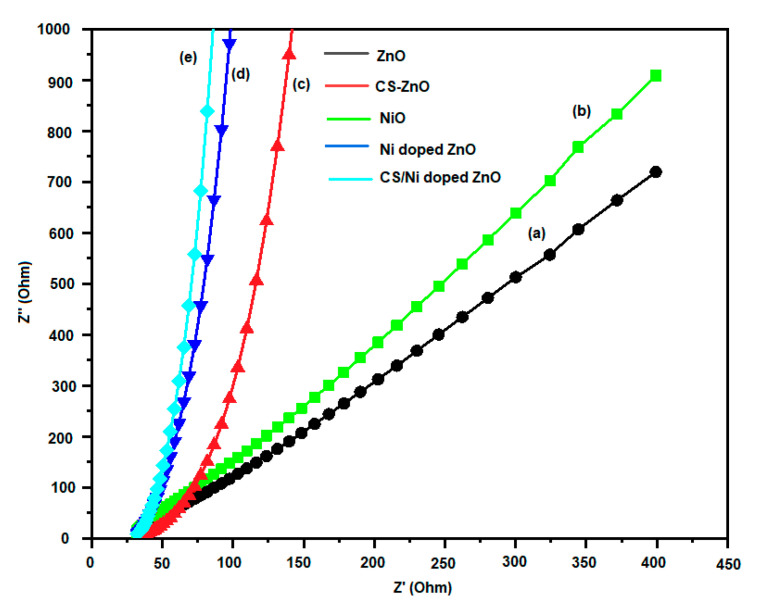
Electrochemical impedance measurements of (**a**) ZnO, (**b**) CS/ZnO, (**c**) NiO, (**d**) Ni-doped ZnO and (**e**) CS/Ni-doped ZnO nanocomposite modified electrodes.

The electrochemical impedance of ZnO, CS/ZnO, NiO, Ni-doped ZnO and CS/Ni-doped ZnO nanocomposite modified electrodes were measured using a 50 mM phosphate buffer solution (PBS) solution containing 5 mM [Fe(CN)_6_]^3−/4−^ in the frequency range of 0.1 Hz to 100 KHz, as shown in Figure 6. Generally, the EIS spectrum consists of a capacitive loop in high frequency and a straight line in low frequency. The capacitive loop is the function of an electron transfer process at the electrode/electrolyte interface, and the straight line in low frequency is associated with the diffusion process. From these results, it can be observed that a straight line denotes Warburg resistance and the diffusion-limiting process, which is due to their good conductivities [59]. The charge transfer resistance values of ZnO, CS/ZnO, NiO, Ni-doped ZnO and CS/Ni-doped ZnO nanocomposite are shown in Table 2. The charge transfer resistance (Rct) value of the CS/Ni-doped ZnO nanocomposite modified electrode is smaller than those of all the other modified electrodes, indicating higher electrical conductivity, which is due to the interactive effect of chitosan and the Ni-doped ZnO. Meanwhile, the cationic chitosan polymer behaves as an electron-conducting mediator in the electron transfer process.

### 3.7. Cyclic Voltammetry Studies

**Figure 7:**
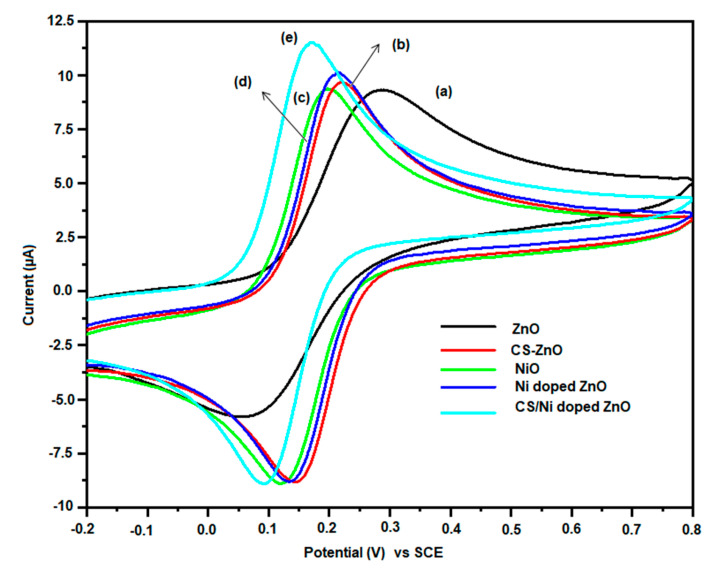
Cyclic voltammograms curves of (**a**) ZnO, (**b**) CS/ZnO, (**c**) NiO, (**d**) Ni-doped ZnO and (**e**) CS/Ni-doped ZnO nanocomposite-modified electrodes.

The cyclic voltammograms of the modified electrodes were performed in the 50 mM PBS containing 5 mM [Fe(CN)_6_]^3−/4−^ with potential from −0.2 to 0.8 V at a scan rate of 50 mV/s, and the results are shown in Figure 7. It can be seen that the CV curves of ZnO, CS/ZnO, NiO, Ni-doped ZnO and CS/Ni-doped ZnO nanocomposite-modified electrodes exhibiting redox peak currents are (9.30/−5.79 µA), (9.33/−8.86 µA), (9.61/−8.74 µA), (10.06/−8.86 µA) and (11.43/−8.87 µA), respectively. It is surprising that the CS/Ni-doped ZnO nanocomposite-modified electrode has a higher redox peak current than those of all the other modified electrodes, indicating that the integration of biogenic chitosan polymer with Ni-doped ZnO nanoparticles can move faster in the electron transfer process between the electrode and electrolyte interface. Also, the synthesized nanocomposite has a higher surface area and mesoporous core–shell structure, which may expose the large electro-active surface area with electrolytes [60]. This result is consistent with the previously discussed BET analysis and EIS studies results.

### 3.8. Antibacterial Activity Mechanism

**Figure 8:**
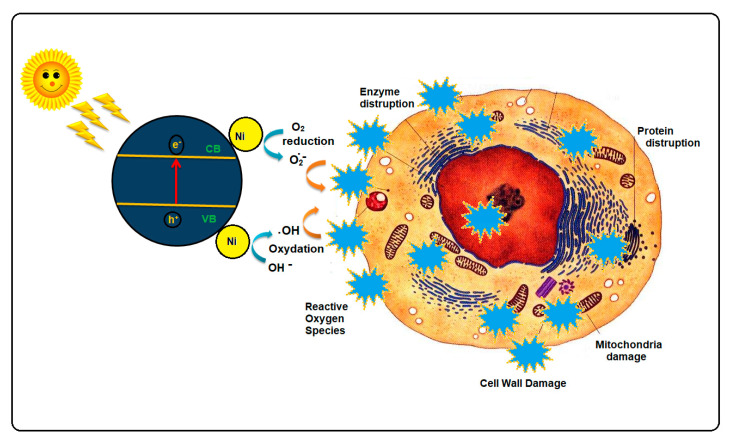
Antibacterial activity mechanism of CS/Ni-doped ZnO nanocomposite.

The proposed antibacterial activity mechanism is explained in Figure 8, where the biogenic chitosan and Ni-doped ZnO synergistically contribute to enhancing the antibacterial and cytotoxicity activities. The antimicrobial activity of the synthesized CS/Ni-doped ZnO nanocomposite was assessed using materials of different concentrations against the listed Gram-positive and -negative strains. Table 3 shows that the antimicrobial activity increased in Gram-positive strains such as *S. aureus*, *L. monocytogens* and *B. subtilis* compared to Gram-negative strains such as *E. coli*, *P. vulgaris* and *V. parahaemolyticus* along with the zone of inhibition.

### 3.9. In Vitro Cytotoxicity

**Figure 9:**
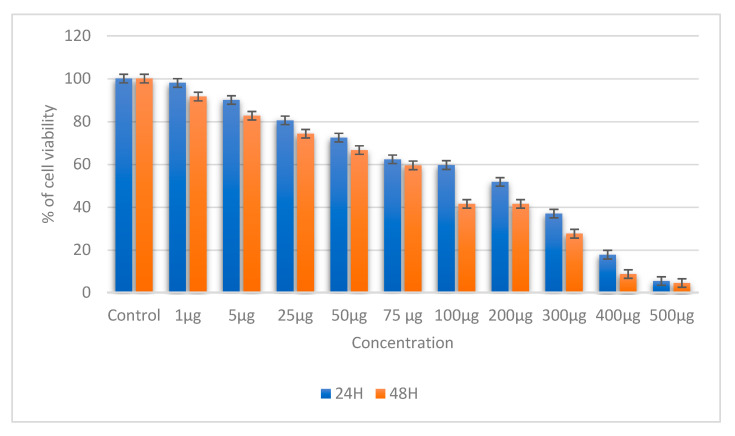
MTT assay of the CS/Ni-doped ZnO nanocomposite in MCF-7 breast cancer cell lines.

**Figure 10:**
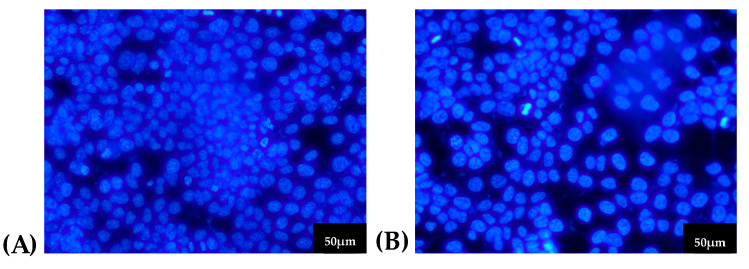
The cytotoxicity effect of the CS/Ni-doped ZnO nanocomposite in MCF-7 breast cancer cell lines. (**A**) control (**B**) treated.

The toxic effect of the CS/Ni-doped ZnO nanocomposite exhibits impressive cytotoxicity with a half-maximum inhibitory concentration of about 80 ± 0.23 µg mL^−1^ against MCF-7 breast cancer cell lines in a dose-dependent manner in an MTT assay (Figure 9). However, the exact mechanism of the CS/Ni-doped ZnO nanocomposite involved in the inhibitory action of MCF-7 breast cancer cell lines is not well understood. Further, the morphology of the MCF-7 breast cancer cells was monitored when treated with the IC50 concentration of the CS/Ni-doped ZnO nanocomposite. In the control experiment, MCF-7 breast cancer cells appeared to have cell blebbing and shrinkage(Figure 10). The primary mechanism is a generation of reactive oxygen species (ROS) on the surface of the nanoparticles, which allows the release of Ni^2+^ and Zn^2+^ ions from the CS/Ni-doped ZnO nanocomposite. When the CS/Ni-doped ZnO nanocomposites are irradiated with light having high photon energy or energy equal to the band gap, they cause the transfer of electrons from the valence band to the conduction band of the synthesized material. The shrinkage of the band gap is caused by nickel doping, which results in an enhancement in the photogeneration of electron-hole pairs [61]. The ability of biogenic chitosan to retard the recombination of photogenerated electron-hole pairs is by readily accepting the free electrons from the conduction band and transporting them quickly through the carbon backbone of biogenic chitosan polysaccharide, which is spread underneath the particles.

It leads to the generation of holes in the valence band, which can react with hydroxyl groups and absorb water to create hydroxyl radicals (−OH) and superoxide anion radicals (O_2-_). The Ni-doped ZnO nanoparticles penetrate through the cell membranes of the bacteria in contact with them [62,63]. The generation of ROS may lead to cell wall dysfunction and rupture due to membrane lipid oxidation [64]. The cationic biopolymer of the chitosan-generated NH^3+^ group (pronoted) electrostatically interacts with the negatively charged pathogenic bacteria cell wall, where positively charged amine groups cause changes in the cell wall membrane structure and produce internal osmotic imbalances to cause cell death [65].

## 4. Conclusions

In this study, we explored the antimicrobial properties of the hybrid CS/Ni-doped ZnO nanocomposite in terms of inhibiting bacterial cell wall growth and inactivating bacterial cells. The characteristics of the CS/ZnO nanocomposite underwent significant changes upon the introduction of Ni particles. The resulting material displayed a uniform rod-like morphology, a well-defined crystal structure, and a specific surface area of 323.9476 m^2^/g. We observed increased UV absorption and reduced band-gap energy, contributing to enhanced antibacterial and anticancer activities. 

In comparison to the CS/ZnO nanocomposite, the CS/Ni-doped ZnO nanocomposite exhibited significantly higher antibacterial activity. This difference can be attributed to the alteration of the basal spacing of ZnO caused by the introduction of Ni, resulting in the generation of a higher number of reactive oxygen species. Notably, the CS/Ni-doped ZnO nanocomposite demonstrated pronounced cytotoxicity with a half-maximum inhibitory concentration of approximately 80 ± 0.23 µg mL^−1^ against MCF-7 breast cancer cell lines, highlighting its potential applications across various fields.

## Figures and Tables

**Table 1:** The results of the BET analysis for ZnO, CS/ZnO, NiO, Ni-doped ZnO and CS/Ni-doped ZnO nanocomposites.

Sample Name	Surface Area (m^2^g^−1^)	Pore Volume (cm^3^g^−1^)	Pore Size (nm)
ZnO	32.2599 m^2^/g	0.064018 cm^3^/g	6.17461 nm
CS/ZnO	45.8004 m^2^/g	0.163342 cm^3^/g	8.60041 nm
NiO	17.5387 m^2^/g	0.013045 cm^3^/g	3.52548 nm
Ni-doped ZnO	75.7213 m^2^/g	0.316305 cm^3^/g	11.67246 nm
CS/Ni-doped ZnO	323.9476 m^2^/g	1.473482 cm^3^/g	18.19407 nm

**Table 2:** The charge transfer resistance values of ZnO, CS/ZnO, NiO, the Ni-doped ZnO and the CS/Ni-doped ZnO nanocomposites.

Sample Name	Rct (Ω)
ZnO	56.64
CS/ZnO	41.14
NiO	57.56
Ni-doped ZnO	29.24
CS/Ni-doped ZnO	18.26

**Table 3:** Zone of inhibition of CS/Ni-doped ZnO against Gram-positive and Gram-negative bacteria.

CS/Ni Doped ZnO	Zone of Inhibition					
Samples	*S. aureus*	*L. monocytogens*	*B. subtilis*	*E. coli*	*P. vulgaris*	*V. parahemolyticus*
25 µl	16 mm	16 mm	17 mm	16 mm	17 mm	12 mm
50 µl	15 mm	17 mm	16 mm	17 mm	16 mm	14 mm
75 µl	15 mm	17 mm	17 mm	17 mm	19 mm	16 mm
100 µl	16 mm	18 mm	18 mm	18 mm	19 mm	17 mm

## Data Availability

All data are included in this article.
